# Cytoplasmic dynein binding, run length, and velocity are guided by long-range electrostatic interactions

**DOI:** 10.1038/srep31523

**Published:** 2016-08-17

**Authors:** Lin Li, Joshua Alper, Emil Alexov

**Affiliations:** 1 Department of Physics, Clemson University, Clemson, SC 29634, USA

## Abstract

Dyneins are important molecular motors involved in many essential biological processes, including cargo transport along microtubules, mitosis, and in cilia. Dynein motility involves the coupling of microtubule binding and unbinding to a change in the configuration of the linker domain induced by ATP hydrolysis, which occur some 25 nm apart. This leaves the accuracy of dynein stepping relatively inaccurate and susceptible to thermal noise. Using multi-scale modeling with a computational focusing technique, we demonstrate that the microtubule forms an electrostatic funnel that guides the dynein’s microtubule binding domain (MTBD) as it finally docks to the precise, keyed binding location on the microtubule. Furthermore, we demonstrate that electrostatic component of the MTBD’s binding free energy is linearly correlated with the velocity and run length of dynein, and we use this linearity to predict the effect of mutating each glutamic and aspartic acid located in MTBD domain to alanine. Lastly, we show that the binding of dynein to the microtubule is associated with conformational changes involving several helices, and we localize flexible hinge points within the stalk helices. Taken all together, we demonstrate that long range electrostatic interactions bring a level of precision to an otherwise noisy dynein stepping process.

Dynein^1^,^2^ is a family of microtubule associated motor proteins consisting of 1–3 heavy chains (>500 kDa) and multiple associated light and intermediate chains^3^. There are two primary subfamilies of dyneins: axonemal dyneins produce the forces that make eukaryotic cilia and flagella beat^4^ and cytoplasmic dyneins produce various movements within cells, including vesicle and organelle transport^5^, mitotic spindle formation^6^ and oscillation^7^, chromosome segregation^8^, and intraflagellar transport^9^. Dysfunctions in dynein lead to multiple debilitating human diseases, including ciliopathies^10^, lissencephaly^11^,^12^ and other neurodegeneration disorders^13^,^14^,^15^, and they can be coopted by viruses during infection^1^.

Cytoplasmic dynein (hereafter, all references to dynein specifically refer to cytoplasmic dynein) contains a homodimer of heavy-chains. Dynein heavy-chains, in turn, consist of several structural domains^16^: a tail domain that is responsible for dimerization and binds cargo as well as light and intermediate chains^17^; a linker domain that reorients during dynein’s powerstroke^18^; six AAA+ (ATPases Associated with diverse cellular Activities) domains that hydrolyze ATP to generate force and drive dynein’s motility^19^; an approximately 15 nm long (as measured from PDB ID 4RH7) coiled coil stalk comprised of coiled coil 1 (CC1) and coiled coil 2 (CC2) that emerges from AAA4 and terminates in the microtubule-binding domain (MTBD)^20^; the MTBD, also called the stalkhead^21^, that binds to microtubules in an ATP dependent manner^5^,^7^,^20^; a strut (or buttress) domain that emerges from AAA5 and interacts with the stalk^22^,^23^; and a C-terminus that regulates motility^24^. All domains after the tail domain collectively form a ~380 kDa minimal motor domain^25^,^26^,^27^.

Dynein moves processively along microtubules in a predominantly minus-end directed manner, as determined by the interaction between the MTBD and the microtubule^7^. Dynein moves by hydrolyzing ATP at the primary catalytic site in AAA1 and converting the released energy into a complex sequence of coordinated conformational changes^16^,^28^,^29^. This involves both the coupling of MTBD binding and unbinding to ATP hydrolysis and the binding of the MTBD to precise locations at the interface between α-tubulin and β-tubulin on the microtubule lattice. The coupling between MTBD binding and unbinding to ATP hydrolysis, which occur some 25 nm apart, is thought to be mediated by intramolecular signaling: a re-registration of the coiled coil stalk^2^ transmits information about the hydrolysis state of the nucleotide bound to AAA1, probably through an AAA3 ATP-dependent manner^4^, to the MTBD, and causes a switching of microtubule affinity^2^,^5^. The MTBD of dynein is in a high-affinity microtubule binding state in the α registry and a low-affinity microtubule binding state in the β+ registry^2^,^5^. Reflexively, re-registration of the stalk upon binding to the microtubule also has been shown to regulate the ATP hydrolysis rate at the AAA1 domain^6^. Binding of the MTBD to precise locations at the interface between α-tubulin and β-tubulin on the lattice, spaced approximately 8 nm longitudinally and 6 nm laterally, requires either extremely precise coordination of ATP hydrolysis and the induced conformational changes or some other mechanism to guide the MTBD binding to the correct binding location. The role of charge interactions to these processes is not fully understood.

Mechanisms for how structural differences between the high-affinity and low-affinity MTBD conformations regulate the MTBD-microtubule affinity have recently been proposed based on structural models, molecular dynamics (MD) simulations, and biophysical characterizations of wild-type and mutant proteins. Both intramolecular^5^ (within the MTBD itself) and intermolecular (between the MTBD and the microtubule)^30^,^31^ salt bridges were proposed to be critical to dynein-microtubule interaction. Specifically, K3298 on H1 of the mouse MTBD was suggested to form a salt bridge that dynamically alternated between E3289 on dynein’s CC1 and glutamic acids on β-tubulin in the microtubule when the MTBD was in the high-affinity conformation^5^. Mutating E3289 to a lysine, which was suggested to bias the interaction toward forming a salt bridge with the glutamic acids on β-tubulin, decreased dynein’s speed and increased its run length^5^. Similarly, R3382 on H6 of the mouse MTBD was suggested to form a salt bridge that switched from E3387 on H6 of the MTBD to glutamic acids on α-tubulin in the microtubule when the MTBD switched from the low-affinity to the high-affinity conformation^5^. Mutating E3378 to a lysine, which was suggested to bias the interaction toward forming a salt bridge with the glutamic acids on α-tubulin, again, decreased dynein’s speed and increased its run length^5^. It was also suggested that yeast α-tubulin’s R403A interacts with E3390 in H1 of the *Dictyostelium* MTBD and α-tubulin’s E416A interacts with R3469 in H6 and K3472 in the H6-CC2 loop of the MTBD^30^. Mutating E415, D417, and E421 in α-tubulin to alanine, a neutral residue, reduced dynein’s run length^30^. Finally, a series of 22 charged amino acids were mutated to alanine on the *Dictyostelium* MTBD^32^. Of the 22 substitutions made, only 6 showed no measurable effect, while one enhanced binding, 11 decreased binding, and 4 slowed nucleotide-stimulated release, further suggesting the possible importance of electrostatics on dynein-microtubule affinity^32^. Together, these studies make it clear that the mechanism of MTBD binding to the microtubule is critical to determining the biophysical properties of dynein. However, it remains unclear how charge interactions directly relate to these properties.

To further understand the role of electrostatics, one could employ atomistic computer modeling. However, the long timescale of stepping events (10s of ms) and the size of the dynein (about 30 nm) and microtubule (>100 nm) system make it impossible to perform an all-atom molecular dynamic simulation. A commonly used strategy to analyze such large systems is to carry out simulations on a small part, or simplified version, of the full system. For example, a recent study showed how conformational changes in the neck linker of kinesin-1 leads to stepping on several tubulin dimers with coarse-grained simulations^33^, and another work used a kinesin and several tubulin dimers in Brownian motion dynamics simulations^34^. Other strategies that have been used are simplified structure models, in which each residue was represented by two beads^35^, and a normal mode analysis with discrete molecular dynamics^36^ that were applied to the investigation of dynein’s stepping mechanism. Similarly, an interpolated elastic network model was used to investigate the mechanism of dynein’s conformational changes^37^ in the motor domain. Together, these coarse-grained models suggest that minor conformational changes induced by the hydrolysis of ATP in AAA1 are amplified by the motor domain, resulting in dynein’s stepping. However, it remains unclear how these conformational changes can result is precise positioning of the MTBD on the microtubule lattice.

Here we address these issues by quantifying the electrostatic component of binding free energy^38^ using a recently developed multi-scale simulation package (MSSP)^39^ from the DelPhi distribution^40^,^41^,^42^,^43^,^44^,^45^. We use the MSSP to overcome the challenges of studying a large system without sacrificing the accuracy of atomic models. Specifically, we quantify the driving force that precisely positions the MTBD domain on the microtubule and relate the binding affinity to the measured run lengths and velocities of cytoplasmic dynein. We use this relation to predict the effect of mutating each glutamic and aspartic acid within the MTBD domain to alanine on the above properties.

## Results

### Dynein and the microtubule have electrostatically complementary binding interfaces

We calculated the pKa of the tubulin-MTBD complex using the DelPhi-pKa webserver (see Methods)^42^,^46^ to investigate the electrostatic features of dynein and tubulin. We found that the tubulin dimer has a net charge of −16 at pH = 7, similar to what was previously reported^47^. We additionally found that the MTBD structure we used, which includes the segments of CC1 and CC2 within PDBID 3J1T and 3J1U, is neutral^42^,^46^.

**Figure 1:**
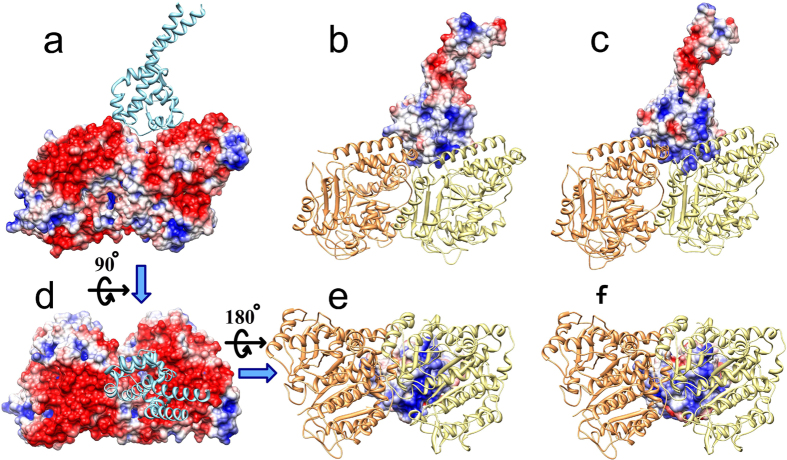
Electrostatic potential mapped onto the MTBD and tubulin dimer. (**a,d**) show the side view and the top view of the electrostatic potential distribution on the surface of tubulin, respectively (filled space, red = negative, blue = positive). (**b,c**) show the side view of the electrostatic potential distribution on the microtubule binding interface of the low- and high-affinity MTBD structures, respectively. (**e,f**) show the bottom view of the electrostatic potential distribution on the microtubule binding interface of the low- and high-affinity MTBD structures, respectively. In (**a,d**) the MTBD (cyan ribbon structure) and in (**b,c,e,f**) the tubulins (α-tubulin, orange ribbon structure and β-tubulin, yellow ribbon structure) are shown for reference, although the potentials are calculated without them.

We calculated the electrostatic potentials of the MTBD and tubulins separately and mapped them onto the surfaces of the tubulin-MTBD structure (Fig. 1 and Supplementary Movie 1). We found that the dynein binding interface on tubulin is strongly negatively charged (Fig. 1a,d). At first, it appears that this negative interface does not strongly influence dynein binding because the MTBD domain is net neutral. However, the charge distribution on the MTBD structure is not homogeneous (Fig. 1b,c), and we found that the binding interface of the MTBD exhibits a strong positive potential in both the high- and low-affinity conformations (Fig. 1e,f). Thus, electrostatic potential is complementary for both the high- and low-affinity conformations. The affinity difference between the high- and low-affinity conformations is likely to be due to the local interactions: several hydrogen bonds and salt bridges^5^ that make the high-affinity conformation to bind stronger to the microtubule.

### Binding free energy and forces guide dynein-microtubule recognition

**Figure 2:**
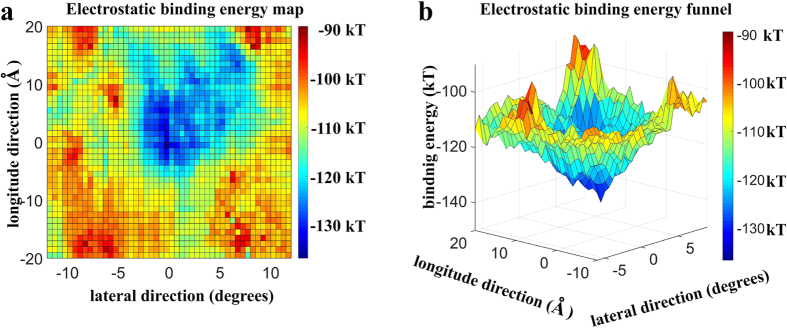
Electrostatic binding energy map of the low-affinity MTBD structure and microtubule. (**a**) 2D representation of the binding energy map in the vicinity of a single tubulin dimer on the microtubule. The reported binding energy (color map) is the binding energy of the most favorable orientation at that particular location for the MTBD on the microtubule surface (see Methods). The magnitude of the binding energy is shown with blue indicating the most and red indicating the least favorable binding energy. (**b**) Detail of the entrance to the binding funnel in a 3D representation using the color map from a. and plotting the magnitude of the binding energy in the vertical axis.

We calculated the electrostatic component of the MTBD-microtubule binding free energy with MSSP (see Methods) to address the role of the electrostatics in tubulin-MTBD recognition. We found that the binding pocket has an asymmetric shape (Fig. 2a). The asymmetrical nature of the pocket not only guides precise MTBD positioning, but also it likely orients the MTBD with respect to microtubule, which contributes to the minus-end directed nature of dynein motility^39^.

Achieving precision binding to the microtubule would be difficult for dynein if performed without a fine guidance mechanism. To probe whether such guidance exists, we plotted the electrostatic component of the binding free energy in 3D (Fig. 2b). We found a binding free energy funnel that guides the MTBD into the binding pocket on the microtubule, represented by the deep blue valley (Fig. 2b). The entrance to the binding funnel is approximately 30 Å wide, and the binding funnel is steep. Thus, once the ATP-stimulated conformational changes in dynein’s motor domain position the MTBD within the proximity of the binding pocket (±15 Å offset and 20 Å above the microtubule surface), electrostatics will provide precision guidance as it docks into its final, favorable position and orientation within the binding pocket.

Note that we performed these calculations on the low-affinity MTBD conformation, which is thought to be the conformational state of the MTBD as it approaches the microtubule^30^.

**Figure 3:**
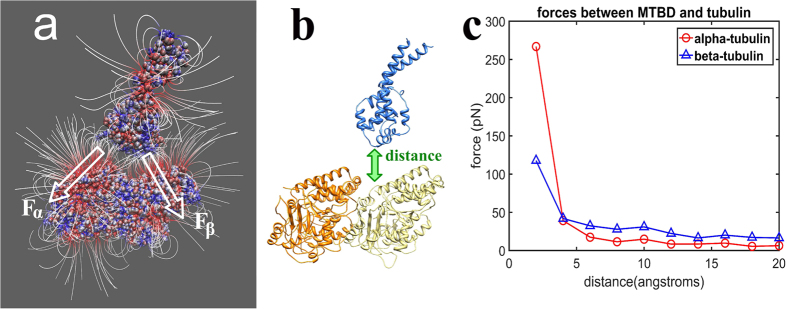
Electrostatic forces between tubulin and the MTBD. (**a**) Electrostatic field lines around and between the MTBD and tubulin dimer calculated with the MTBD separated from tubulin by 20 Å. Proteins are shown as space filling, and the red color indicates negative polarity and the blue color indicates positive polarity. The white arrows, F_α_ and F_β_, indicate the total electrostatic forces between the MTBD and the α- and β-tubulin, respectively. (**b**) Diagram of the distance between the MTBD (blue) and tubulin (orange and yellow), which is varied from 4 to 20 Å with respect to original crystallographic position of MTBD on microtubule. (**c**) The magnitude of the electrostatic forces between α-tubulin and the MTBD (circles, red) and β-tubulin and the MTBD (triangles, blue) as a function of the distance between the MTBD the tubulin.

To further elucidate the role of electrostatics in dynein-microtubule recognition, we investigated the electrostatic field distribution (see Methods) between the MTBD and microtubule (Fig. 3a), separated by a distance of 20 Å. We calculating the total electrostatic force (see Methods) between the MTBD and α- and β-tubulin as a function of the distance between MTBD and microtubule (Fig. 3c). Note that the zero distance point corresponds to crystallographic position, and the distance represents a translation of the MTBD with respect to tubulins (Fig. 3b). We found that these are long-range forces, providing non-negligible guidance even at 20 Å distance.

**Figure 4:**
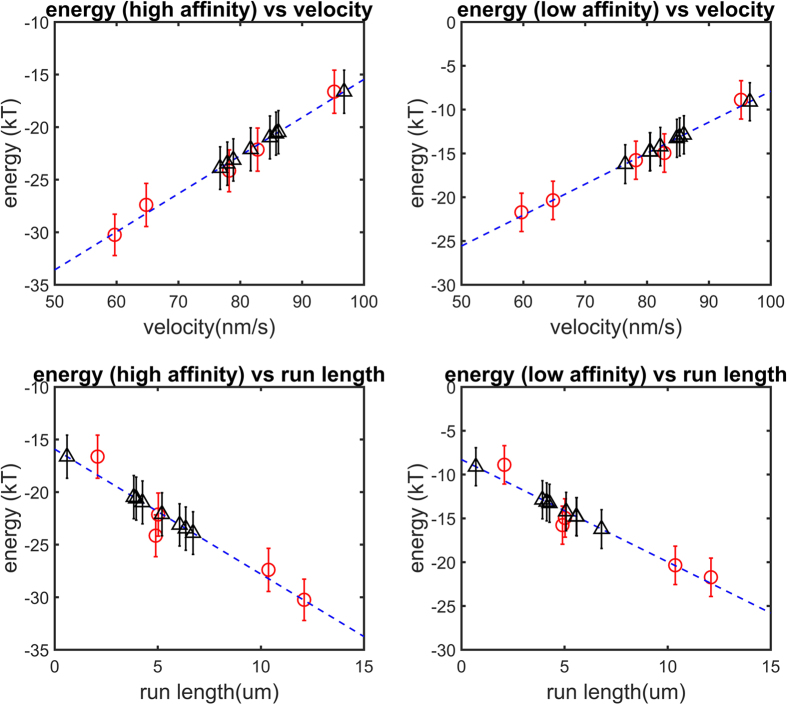
The relationship between electrostatic binding energy and velocity and run length for both high-and low-affinity configurations. In each panel, the mean calculated electrostatic binding energy is plotted as a function of the mean velocity and run length of the respective mutant (five red circles; n = 50 representative structures; error bars represent the standard error of mean; blue dash line is the least square regression linear fit to the five red circles). Eight additional charged residues were mutated *in silico* to Ala (Methods), and their corresponding binding energies were calculated and plotted to predict the mean velocity and run length of the respective mutant (black triangles; error bars represent the standard error of mean).

We estimated the work needed to detach the MTBD from the tubulin using the calculated forces as a function of the distance. We calculated the area below the force-distance curves (Fig. 3c), assuming a small angle between the force and displacement. Thus, by equating work and energy, the energy of separating the MTBD and α-tubulin is 50 pN·nm (or 12 kT); and the energy of separating MTBD and beta tubulin is 55 pN·nm (or 13 kT). Therefore, the work needed to detach the MTBD from tubulin is estimated to be 25 kT, which agrees with the energy calculations (Fig. 4). Note that the assumption of a small angle between the force and the displacement of MTBD-tubulins causes an overestimate of the work, thus the actual energy could be less than 25 kT.

### Dynein run length and velocity are linearly related to the electrostatic component of the binding energy

We investigated the relationship between the electrostatic binding energy and the velocity and run length for the wild type and 4 mutants (E3289Q, E3289K, E3378Q, and E3378K) of the *Mus musculus* cytoplasmic dynein. The mutations are all of charged residues within the MTBD and they are remote from the binding interface^5^. We used an *in silico* mutant preparation to make the mutants (see Methods) and calculated the electrostatic binding energy (see Methods) for 50 representative structures of the tubulin-MTBD dimer complexes obtained from MD simulations (see Methods). We compared the results of the electrostatic binding energy calculations with experimentally measured biophysical quantities (velocity and run length) and performed a least squares linear regression on the data (Fig. 4, Table 1).

We found a strong linear relationship between the electrostatic binding energy and both velocity and run length (Fig. 4 and Table 1) for these MTBDs: the larger the effect of long-range electrostatic interactions between the MTBD and the microtubule on binding energy, the slower a dynein moves and the farther it walks before falling off the microtubule.

The electrostatic component of the binding affinity is linearly correlated with the run length and velocity over a −15 to −35 kT electrostatic binding energy range for the high-affinity conformation and over a −5 to −25 kT electrostatic binding energy range for the low-affinity conformation. We expect this linear relation to hold for other mutations that result in an electrostatic binding energy within this range. Therefore, we made *in silico* mutations to all the glutamic and aspartic acids within the MTBD domain remote from the binding interface and predicted their effect on run length and velocity (Fig. 4 and Table 2). We found that that calculated electrostatic component of the binding energy for all mutants was within the linear range for both the high- and low-affinity structures.

### Dynamic salt bridges do not appear to regulate dynein-microtubule affinity

We used the MD simulation trajectories to investigate the previously suggested potential dynamic salt bridges made between lysine and arginine residues and E3289 and E3378, respectively, within the MTBD^5^. Using the standard salt bridge threshold of 4 Å for the distance between oxygen and nitrogen atoms^48^, we found that neither K3298 and E3289 nor K3298 and E420 on β-tubulin form salt bridges (Supplementary Fig. s1). Changes in the conformational from the crystal structure, which occur during the MD simulation, result in both an elongation of CC1 and an increase in the distance between H1 in the MTBD and β-tubulin, preventing these salt bridges (Supplementary Fig. s1). Additionally, we found no salt bridge between the K3382 and E3378 because K3382 is always attracted by a group of three negatively charged residues (E414, E417, and E420) on α-tubulin; in most structures, K3382 formed salt bridges with either E417 or E420 on α-tubulin (Supplementary Fig. s1).

### The hinge-bending of stalk helices

**Figure 5:**
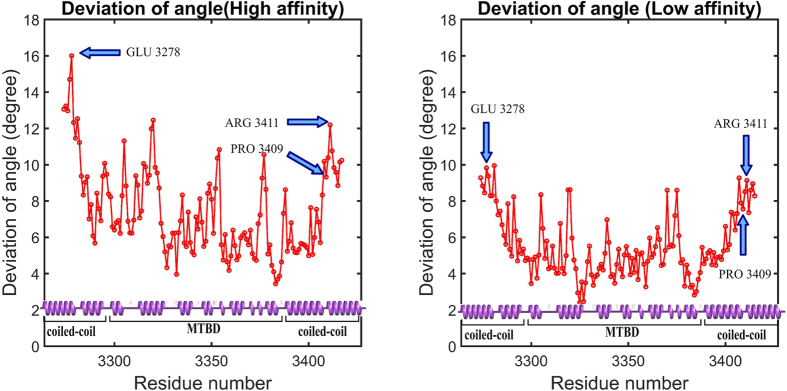
HingeDetector identified E3278 and R3411 as the hinge points in the coiled coil stalk in mouse cytoplasmic dynein. The deviation of angle is the standard deviation of the segment-angles as calculated for all structures in the 5 ns of simulations calculated. The first 10 and last 10 residues of the structure cannot be taken into account, because the segment length was selected to be 10 residues long.

Recent work on a *Dictyostelium discoideum* cytoplasmic dynein found that the coiled coil stalk helices undergo hinge-like bending as dynein walks along microtubule, and it suggested that the hinge occurs at two highly-conserved proline residues (P3371 and P3496) within the stalk helices^21^. Here we investigate whether this suggestion holds for *Mus musculus* cytoplasmic dynein, which also has two proline residues (P3286 and P3409 on CC1 and CC2, respectively) near the MTBD. Using HingeDetector, a novel VMD^49^ software tool that we designed for this purpose (see Methods), we analyzed the conformations of 25,000 structures taken from our MD simulations. We found that the largest standard deviation in the segment angle was associated with residues E3278 and R3411 on coiled coil stalk helix CC1 and CC2, respectively (Fig. 5). The R3411 is only 2 residues away from P3409, and therefore the hinge may be partially attributed to P3409 as was previously suggested^21^. However, the other hinge point, at E3278, is not associated with any neighboring proline. These results suggest that the hinge in CC1 was induced by the hinge in CC2 via inter-helical interactions. That E3278 and R3411 are the hinge points holds for both the high- and low-affinity structures, but the hinge is more flexible in the high-affinity structure (Fig. 5). This suggests that the hinge, which was clearly observed in the microtubule bound state^21^, is activated by the changes associated with the switch from the low-affinity to the high-affinity conformation of the coiled coil stalk.

## Discussion

**Figure 6:**
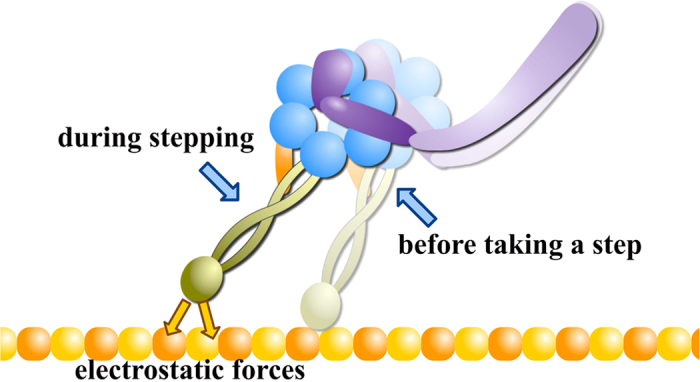
Illustration of the interplay between ATP-hydrolysis induced conformational changes and role of electrostatic forces in dynein’s stepping. For simplicity, this figure only shows two states of a dynein monomer during the stepping process. In the stepping process, electrostatic forces play important role in the final alignment of the MTBD binding as it docks to the correct binding position.

In this work, we further clarified the role of electrostatics in dynein-microtubule recognition. The first key finding is the existence of an electrostatic funnel that guides the MTBD to the final binding spot on the microtubule. This provides insight into the accuracy of the conformational changes triggered by ATP hydrolysis and the walking process of dynein along microtubule (Fig. 6). The coiled-coil stalk (shown in green in Fig. 6) amplifies the conformational changes of the linker (shown in purple in Fig. 6) and moves the MTBD toward its next binding site as dynein steps^7^. Dynein is not an accurate stepper. It randomly takes off-axis and non-alternating steps^50^,^51^, which is likely due to errors both in the motion of the linker domain and due to thermal fluctuations that are amplified by the long coiled coil stalk. Our calculations show that electrostatic interactions provide precise guidance to the MTBD in the final stages of a step as it docks to the biding site once it is in the proximity of the binding pocket. Additionally, the asymmetry in the shape of the binding pocket on the microtubule and in the charge distribution on the MTBD help to orient the dynein to the microtubule, effectively keying its binding orientation and contributing to its directional bias. This electrostatic field mechanism makes the stepping process robust and enables dynein to overcome its otherwise inaccurate tendencies. (Supplementary Movie 2 and Fig. 6).

The second key finding is that the magnitude of the electrostatic component of the binding energy is linearly correlated with dynein’s run length and velocity. We showed how stronger binding energies, which lead to a more stable complex, result in a lower velocity and longer run length of dynein. This is reasonable from physical intuition: it is difficult to walk quickly on a sticky road because the sticky surface slows down the steps, but it is also more difficult to fall off it. We used this correlation to predict the effect of glutamic and aspartic acid mutations to alanine on the run length and velocity of cytoplasmic dyneins. It should be pointed out that in the limits of very strong binding (dynein firmly attached to microtubule) and very weak binding (dynein almost not bound to microtubule) this is not expected to hold.

In the process of making these two key discoveries, we were able to shed additional light on a couple previous observations. First, we note that there are significant differences between salt bridge observations of our work and a previous study^5^. These are likely to be due to differences between the constraints applied to the MD simulations in the studies^5^. In our simulation, the entire MTBD structure, except 4 bonds we introduced on the coiled coil stalk (see Methods), is set free without any constraints. In the previous study^5^, additional constraints were applied by their cryo-EM density maps. Our constraints both let the MTBD sample more conformations and make it less compact. These effects allowed us to explore additional conformational space in the context of the tubulin-MTBD complex, and we found that this additional flexibility precluded some previously hypothesized dynamic salt bridges. Additionally, it suggests that the observed effects of the mutations on dynein motility are primarily governed by differences in long-range electrostatic interactions. Second, we developed a new tool, the HingeDetector, which we used to identify the hinge bending point on the stalk helices. We showed that the hinge bending points are not necessarily associated with prolines, as it was suggested in case of *Dictyostelium*’s tubulin-MTBD complex^21^, but are the result of the presence of neighboring proline and inter-helical interactions.

Taken all together, we demonstrate that long range electrostatic interactions bring a level of precision to an otherwise noisy dynein stepping process. While it is likely that certain salt-bridges at the interface between the microtubule and the MTBD may regulate affinity once docked, when the residues are close enough to form these bridges, there is a considerable contribution made by long-range electrostatic interactions getting the MTBD close enough in the first place to make these bonds. And these effects are sufficient to explain much of variability in velocity and run length when mutations are made to charged AAs in the MTBD.

## Methods

### Structure preparation

#### Tubulin-MTBD complex structures

**Figure 7:**
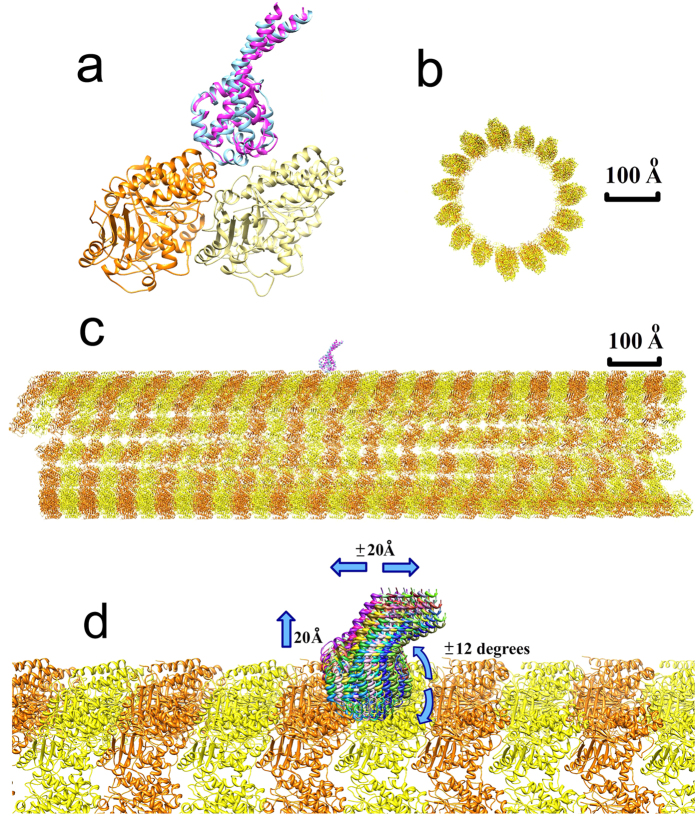
Structures and sampling strategy used in MSSP computations. (**a**) Structure of the tubulin-MTBD complex, consisting of an α-tubulin (orange), a β-tubulin (yellow), and either a low-affinity MTBD (blue) or a high-affinity MTBD (pink). (**b**) Cross-sectional view of the microtubule structure. (**c**) The side view of the microtubule structure with MTBDs on the top. (**d**) Multiple MTBD positions (various colors) with respect to the microtubule. For clarity, only a fraction of the generated positions is shown. The blue arrows indicate that the MTBD position was varied within a ± 20 Å window along the microtubule, up to 20 Å above the microtubule surface, and within a ± 12 degree window around to the microtubule.

The tubulin-MTBD complex structures used in the MD simulations and for placing the MTBD onto a large-scale model of microtubule were *Bos taurus* tubulin and *Mus musculus* cytoplasmic dynein. The specific MTBD structures used were a low-affinity conformation (PDB ID 3J1U) and a high-affinity conformation (3J1T)^5^ (Fig. 7a). The high- and low-affinity MTBD structures are quite similar, with the largest difference observed at the tubulin-MTBD binding interface.

#### Microtubule structure

A large piece of microtubule structure was generated and used in the MSSP to calculate the long-range electrostatic potential, field, and binding energy of the MTBD-microtubule complex. Rotation and translation matrices (PDB ID 3J2U)^52^ were used to model a 16-dimer long segment of a microtubule (total length = 1280 Å and diameter = 300 Å; Fig. 7b,c). The tubulin-MTBD complex structures (above) were aligned to the microtubule structure to determine the binding position of each MTBD configuration on a microtubule (Fig. 7c). The tubulin structure used for this alignment was taken from a higher resolution crystal structure (PDB ID 1JFF)^53^.

### Electrostatic potential, binding energy, and field line calculations

The 3D distribution of the electrostatic potential was modeled with DelPhi^40^,^41^,^42^,^43^,^44^,^45^,^54^, which calculates the electrostatic potential by solving the Poisson-Boltzmann equation (PBE). The parameters used in the calculations were: the scale, which was set at a resolution of 2 grid/Å; the perfil (percentage of the protein that occupies the grid box), which was set at 70; the reference dielectric constants, which were set at 2 for the protein and 80 for the water. The boundary condition used was the “dipole boundary condition” and the force field parameters were taken from CHARMM^55^. The salt concentration was set at zero to avoid any possible ambiguity due to the explicit ion binding effect, which is expected to occur in such highly charged systems^56^. The calculated electrostatic potential was used to generate electrostatic field lines (using VMD^49^) and to calculate the electrostatic component of the binding energy of the MTBD to the tubulin. Note that when we present the charge complementarity between the MTBD and the microtubule (Figs 3, 5 and 6), we only consider the tubulin-MTBD complex so that the rest of the microtubule would not obscure the key details. However, the binding energy profile (Fig. 2) was calculated using the entire microtubule and MTBD.

Based on the electrostatic potential obtained by solving the PBE, the corresponding electrostatic energies (Coulombic and reaction field energies) were calculated as previously described^57^). The electrostatic component of binding energy was calculated as in equation (1):


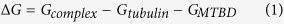


where Δ*G* is the electrostatic component of binding energy of the tubulin-MTBD complex; *G*_*complex*_ is the electrostatic energy of the complex; *G*_*tubulin*_ is the electrostatic energy of the tubulin structure; and *G*_*MTBD*_ is the electrostatic energy of MTBD structure.

### Binding energy profile generation by MSSP

A multi-scale simulation package (MSSP) was used to generate the binding energy profile of the MTBD-microtubule complex, as was previously described and used to model kinesin binding to a microtubule^39^,^45^. Briefly, three steps were taken to generate the binding energy profile in the MSSP: 1) the sampling module used cylindrical sampling to generate an ensemble of MTBD positions around a piece of the microtubule (Fig. 7d) with steps of 0.5 Å in the longitudinal and normal directions, and steps of 0.5 degrees around the microtubule. In addition, the MTBD was to sample possible orientations as 9 rotamers were generated to model the possible orientations of the MTBD at each position by searching in the Euler space^39^. The generated orientations do not explore the full Euler space because the MTBD’s binding interface is constrained to face the microtubule and the coiled-coil stalk is constrained to point to the water phase by the rest of the dynein structure. In total, 80 ×48 ×40 × 9 = 1,382,400 MTBD positions were generated by the sampling module. 2) The energy module calculated the binding energy for each pre-generated position of the MTBD. Since the MSSP utilized a rigid body protocol, i.e. the MTBD and microtubule structures were unchanged, there was no need to calculate mechanical energy. Thus, the binding energy in MSSP was composed of the electrostatic and van der Waals energies only. The electrostatic energy was calculated with a computational focusing method, which significantly saves on calculation time while taking into account the entire microtubule^39^,^42^. 3) The energy module collected the binding energies for all the sampled positions of the MTBD and converted the binding energies from a 4-dimensional array into a 2-dimensional binding energy profile map to reduce the complexity and allow for visualization. For each MTBD position along and around the microtubule, the binding energy that was recorded and visualized corresponded to the normal position and angular orientation with the lowest binding energy because this represents the highest probability normal position and angular orientation.

### Molecular dynamics simulations

Molecular dynamic (MD) simulations were performed on the high-and low-affinity tubulin-MTBD conformations, not the microtubule model because of its large size, using NAMD^58^. The tubulin-MTBD complex represents a small fraction of entire dynein-microtubule complex. Therefore, neither the MTBD nor the tubulin are independent of the rest of the dynein or microtubule structures. To account for this, the tubulin residues that are not at the tubulin-MTBD interface (those residues >10 Å from any atoms on MTBD, Supplementary Fig. s2) were constrained using a harmonic constraint energy function with a scaling factor of 1.0. Additionally, four bonds with spring constants of 100 kcal/mol/Å^2^, which is the approximate strength of a C-C bond, were added to the coiled-coil stalk of dynein to mimic the stabilization imparted by the missing part of the full dynein structure (Supplementary Fig. s2). These extra bonds were added to the C_α_ atoms of residue pairs of Q3265-N3424, I3269-A3421, Q3273-L3419, and V3276-L3414. All other residues were unconstrained in the MD simulations. The MD simulations were carried out for 5 nanoseconds, and the structures at the end of the 5 nanosecond simulations were used as high-and low-affinity reference structures. For computations that require multiple representative structures, another 5 nanoseconds of simulation were carried out, and snap-shots were selected every 0.1 nanoseconds.

### *In silico* mutant preparation

Mutations within the high- and low-affinity reference structures and corresponding snapshots, obtained above, were generated using profix^59^. No attempt was made to relax the mutant structures to emphasize the role of electrostatics and minimize the effect of structural changes on the binding energy calculations. This was done because all mutation sites are away from the binding interface, suggesting that the major differences in the calculated binding energies between mutants and wild type are expected to be electrostatic. The electrostatic component of the binding energy for each mutant structure was calculated by DelPhi, and the results were averaged over 50 snapshots.

### Hinge bending localization

**Figure 8:**
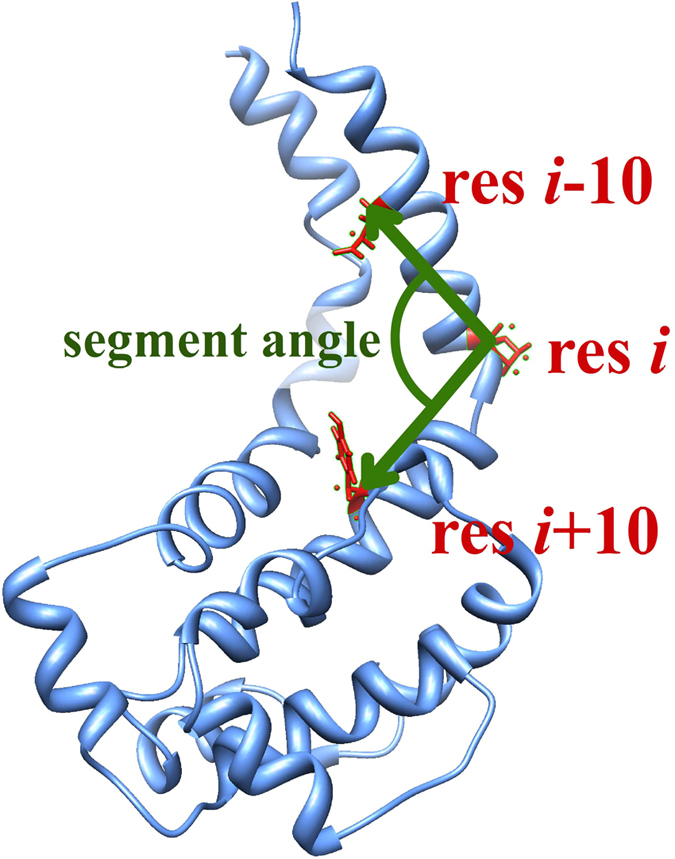
Illustration of HingeDetector algorithm. HingeDetector generates two adjacent structural segments of length 10 residues defined by residues *i*-10, *i*, and *i*-10 (red). It slides these structural segments over the protein (blue) in one residue steps and calculates the segment angle between the vectors (green) formed by the structural segments. The residue, *i*, with the largest standard deviation in segment angle calculated over all snap-shots is identified as a hinge point.

A hinge detection tool, HingeDetector, was developed and presented here to identify the hinge residues in a MD simulation trajectory. For each structure in the trajectory, HingeDetector calculates the angle, called the segment angle, between two vectors: vector one is defined by the line between the C_α_ atom of residue *i* and the C_α_ atom of residue *i*-10, and vector two is defined the line between the C_α_ atom of residue *i* and the C_α_ atom of residue *i* + 10 (Fig. 8). Note that multiple window lengths were explored, but the 10 residue window optimized the detection of global changes involving large structural segments. Then, HingeDetector calculates the standard deviation of the segment angle for position *i* based on provided snapshots and moves to the next position, *i* + 1. The greater the standard deviation, the more flexible are the structural segments associated with position *i*, therefore the residue with the highest deviation in segment angle is identified as a hinge point. This HingeDetector tool is designed as a tool in VMD^49^, and it is available on our website^60^. Alternatively, one can use the recently developed multiscale method to detect hinge points^61^.

### Electrostatic force calculations and evaluation of the work needed to detach MTBD from tubulin

Delphi was used to calculate the total electrostatic force between the MTBD and the tubulins using the FRC module^62^. The MTBD was provided as an FRC file, and the charges of all MTBD atoms were turned off. Then, DelPhi calculated the electric field generated by tubulins at the positions of each atom in the MTBD. The electric field acting on each MTBD atom was multiplied by the corresponding charge of that atom, which resulted in the force on each MTBD atom. The total force on the MTBD was calculated by summing the forces on all of the MTBD atoms. This process was repeated for normal displacements of the MTBD up to 20 Å from the bound position in 2 Å increments. The forces were numerically integrated over the increasing distance between the MTBD and the tubulins to calculate the work needed to detach the MTBD from tubulin.

## Additional Information

**How to cite this article**: Li, L. *et al.* Cytoplasmic dynein binding, run length, and velocity are guided by long-range electrostatic interactions. *Sci. Rep.* **6**, 31523; doi: 10.1038/srep31523 (2016).

## Supplementary Material

Supplementary Movie 1

Supplementary Movie 2

Supplementary Information

## Figures and Tables

**Table 1:** Linear regression results (electrostatic binding energy = slope (velocity OR run length) + intercept).

	Slope	Intercept	Correlation coefficient (R)
High-affinity, velocity	0.36 kT s/nm	−52 kT	0.91
High-affinity, run length	−1.2 kT/μm	−16 kT	−0.87
Low-affinity, velocity	0.35 kT s/nm	−43 kT	0.90
Low-affinity, run length	−1.2 kT/μm	−8.3 kT	−0.88

**Table 2:** Predicted velocities and run lengths for investigated mutations.

Mutations	Electrostatic binding energy (high-affinity) (kT)	Electrostatic binding energy (low-affinity) (kT)	Velocity predicted from high-affinity calculations (nm/s)	Velocity predicted from low-affinity calculations (nm/s)	Run length predicted from high-affinity calculations (μm)	Run length predicted from low-affinity calculations (μm)	Velocity averaged from high-and low-affinity calculations (nm/s)	Run length averaged from high-and low-affinity calculations (nm/s)
D3332A	−23	−15	78	80	6.4	5.6	79	6.0
D3359A	−20	−13	86	86	3.8	3.9	86	3.9
E3320A	−24	−15	77	81	6.7	5.6	79	6.1
E3328A	−22	−14	82	82	5.2	5.1	82	5.1
E3343A	−23	−16	79	76	6.1	6.8	78	6.4
E3356A	−21	−13	85	85	4.3	4.3	85	4.3
E3363A	−21	−13	86	85	4.0	4.1	86	4.0
E3402A	−17	−9.1	97	97	0.60	0.70	97	0.65
