# Supplementary material for: Cytoplasmic dynein binding, run length, and velocity are guided by long-range electrostatic interactions
Source: Sci Rep. 2016 Aug 17;6:31523. doi: 10.1038/srep31523 (PMC4987762; doi:10.1038/srep31523)
Supplement: Supplementary Information [file srep31523-s3.pdf]

Cytoplasmic dynein binding, run length, and velocity are guided by long-range electrostatic interactions

Lin Li[1], Joshua Alper[1,*], Emil Alexov[1,*]

[1] Department of Physics, Clemson University, Clemson, SC 29634, USA

* alper@clemson.edu, ealexov@clemson.edu

Supplementary Figures:

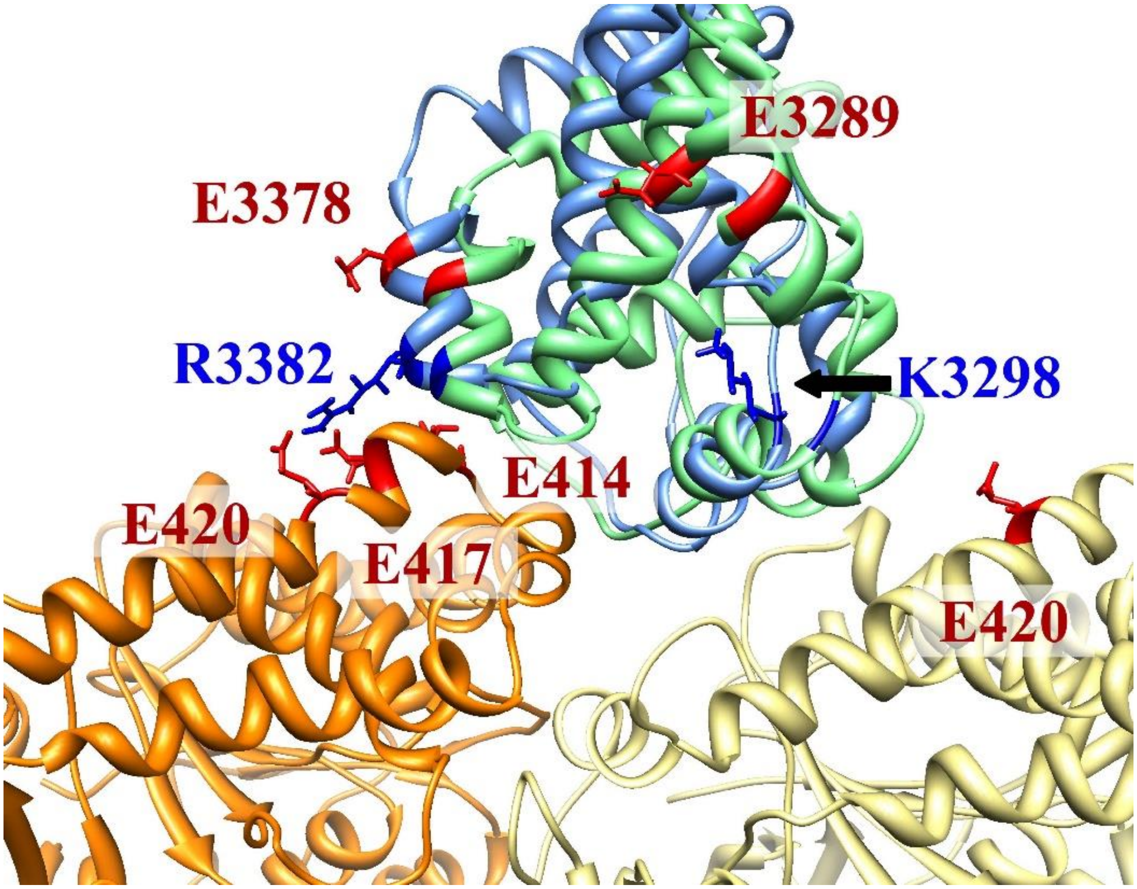

Figure S1. The distribution of charged residues close to E3378 and E3289 in the MD simulation. α-tubulin is in orange; β-tubulin is in yellow. The high affinity MTBD structure in the MD simulation is in blue and the MTBD structure of crystal structure is in green. In the MD simulation, no salt bridge is detected between either K3298-E3289 or K3298-E420 (β-tubulin). Additionally, there are no salt bridges between R3382 and E3378 despite the apparent proximity of R3382 to E3378 because the 3 noted glutamic-acid residues on α-tubulin hold it so strongly it cannot escape. In most structures in the simulations, R3382 formed salt bridges with either E417 or E420 (α-tubulin).

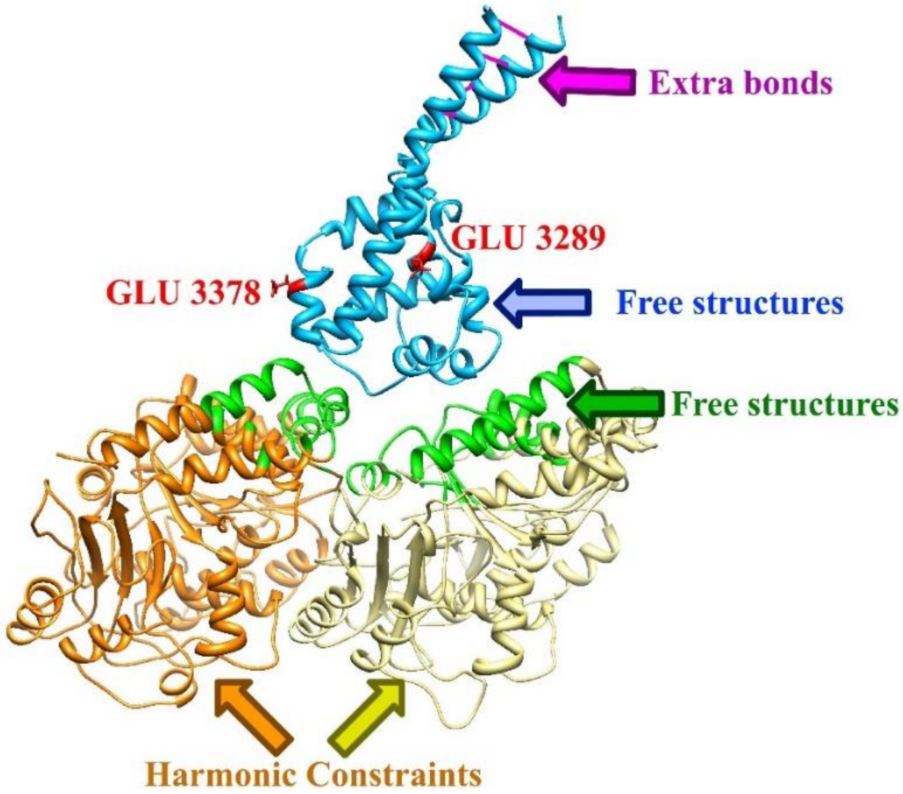

Figure S2. Preparation for MD simulations. 4 extra bonds (magenta) are added to the coiled-coil structure; Harmonic constraints are performed on the non-interfacial residues of tubulin (orange and yellow); all the other structures (green and blue) are free in the MD simulation. The two mutation positions are labeled in red.

Supplementary Video Captions:

Movie 1. An illustration of electrostatic potential on the surfaces of the microtubule binding domain and tubulin.

Movie 2. An animation of one step for dynein moving along microtubule.
